# Increased hippocampal GABAergic inhibition after long-term high-intensity sound exposure

**DOI:** 10.1371/journal.pone.0210451

**Published:** 2019-05-08

**Authors:** Alexandra O. S. Cunha, Junia L. de Deus, Cesar C. Ceballos, Ricardo M. Leão

**Affiliations:** Department of Physiology, FMRP, University of São Paulo, Ribeirão Preto, SP, Brazil; McGill University, CANADA

## Abstract

Exposure to loud sounds is related to harmful mental and systemic effects. The hippocampal function can be affected to either high-intensity sound exposure or long-term sound deprivation. We previously showed that hippocampal long-term potentiation (LTP) is inhibited after ten days of daily exposure to 2 minutes of high-intensity noise (110 dB), in the hippocampi of Wistar rats. Here we investigated how the glutamatergic and GABAergic neurotransmission mediated by ionotropic receptors is affected by the same protocol of high-intensity sound exposure. We found that while the glutamatergic transmission both by AMPA/kainate and NMDA receptors in the Schaffer-CA1 synapses is unaffected by long-term exposure to high-intensity sound, the amplitude of the inhibitory GABAergic currents is potentiated, but not the frequency of both spontaneous and miniature currents. We conclude that after prolonged exposure to short periods of high-intensity sound, GABAergic transmission is potentiated in the hippocampal CA1 pyramidal neurons. This effect could be an essential factor for the reduced LTP in the hippocampi of these animals after high-intensity sound exposure. We conclude that prolonged exposure to high- intensity sound could affect hippocampal inhibitory transmission and consequently, its function.

## Introduction

Exposure to loud noises is related to several deleterious mental and systemic effects [1,2], in addition to auditory maladies as deafness, hyperacusis, and tinnitus [3,4]. In fact, loud sounds from both occupational and recreational sources became a common occurrence for several people, and health problems related to sound exposure are increasingly common, even in juveniles [5,6,7]. However, while the effects of high-intensity sound exposure in the central auditory neurons have been extensively studied, the consequences to other brain areas, especially in areas related to cognition and emotions, are less known.

The hippocampus is a region traditionally implicated in the formation of declarative and spatial memories and is connected to the auditory system indirectly from the frontomedial cortex, insula, and amygdala [8], and directly by a recently identified contact between the auditory cortex and CA1 area [9]. This pathway is implicated in the formation of long-term auditory memories [10], and acoustic cues can be used in the formation of spatial memories [11]. Additionally, hippocampal place cells can be activated by a task using auditory dimension cues [12] and recently it was demonstrated that sound stimulation evokes excitatory and inhibitory currents in CA3 pyramidal neurons *in vivo* [13].

Accordingly, the hippocampus is affected by sound stimulation or sound deprivation. Prolonged (2 hours/ day for 3–6 weeks) moderate (80 dB) sound exposure, impairs spatial memory in mice and increases oxidative damage and tau phosphorylation in the hippocampus [14,15]. Acute traumatic noise (106–115 dB, 30–60 minutes) alters place cell activity in the hippocampus [16] and increases *arc* expression, an immediate early gene related to synaptic plasticity, in the hippocampi of rats [17]. Work from our group has shown that long-term (10 days, 2 minutes a day) stimulation with high-intensity broadband sound inhibits LTP in the Schaffer-CA1 hippocampus of rats [18] and hyperpolarizes CA1 pyramidal neurons by decreasing the expression of the h current (I_h_), while increasing the firing of these neurons by an unknown ionic mechanism [19]. The effect on LTP, but not on the membrane intrinsic properties of CA1 neurons, was also observed after a single one-minute 110 dB broadband sound stimulus [20]. In order to investigate the possible mechanisms underlying the inhibition of LTP by high-intensity sound, we studied, using whole-cell patch-clamp recordings *in vitro*, the excitatory and inhibitory neurotransmission in CA1 pyramidal neurons from animals subjected to long-term exposure to episodes of high-intensity broadband noise.

## Material and methods

### Animals

All experimental procedures involving animals were designed in strict accordance with the recommendations for animal research from the National Council for Animal Experimentation Control (CONCEA), and this study was approved by the Commission for Ethics in Animal Experimentation (CEUA) at the University of São Paulo at Ribeirão Preto (protocols 015/2013 and 006/2-2015). All efforts were made to minimize animal suffering.

Male Wistar rats (60–80 days) were obtained from the central animal facility of the University of São Paulo-Ribeirão Preto Campus and kept in the rat animal facility of the School of Medicine of Ribeirão Preto until the day of use. The animals were kept in Plexiglas cages (2–3 animals per cage), food and water *ad libitum* and 12-h dark/light cycle (lights on at 7:00 a. m.) and controlled temperature (22°C).

### Sound stimulation protocol

Our protocol was previously described in [18]. Briefly, animals were placed in an acrylic arena (height: 32 cm, diameter: 30 cm) located inside an acoustically isolated chamber (45 x 45 x 40 cm, 55 dB ambient noise) where, after one minute of habituation, they were submitted to a one-minute episode of 110-dB sound stimulus (a digitally modified recording of a doorbell, spanning frequencies from 2 to 15 kHz, with a peak at 7 kHz) [21]. The animals were kept in the stimulation chamber for one more minute and then returned to their home cages. In case the animals presented seizures [18], they were discarded from the study. This protocol was repeated for ten days, twice a day (8–9 am and 4–5 pm). The animals rested for 10–14 days after the last session before sacrifice. The control group was placed in the box for the same amount of time and not subjected to sound stimulation.

### Hippocampal slices

Animals were anesthetized with isoflurane, decapitated and the brains rapidly removed and placed in an ice-cold solution containing (mM): 87 NaCl, 2.5 KCl, 25 NaHCO_3_, 1.25 NaH_2_PO_4_, 75 Sucrose, 25 Glucose, 0.2 CaCl_2_, 7 MgCl2_,_ bubbled with 95% O2 and 5% CO_2_. The brain hemispheres were separated and fixed with cyanoacrylate glue to a support and placed inside the cutting chamber of a vibratome (1000 plus, Vibratome, USA) filled with the same solution and cut in 200 μm transverse slices containing the dorsal hippocampus. Then, the slices were placed in aCSF solution containing (mM): 120 NaCl, 2.8 KCl, 1.25 NaH_2_PO_4_, 26 NaHCO_3_, 20 Glucose, 2 CaCl_2_, 1 MgCl_2_ at 34–35°C for 45 min. Slices were then left at room temperature until use.

### Whole cell patch clamp recordings

CA1 pyramidal neurons were visualized with an Olympus BX51WI Microscope (Olympus, Japan) with infrared differential interference contrast (IR-DIC). Neurons were chosen based on the morphology (pyramidal shape) and position in the pyramidal layer. Patch clamp recordings were performed using a Heka EPC10 (HEKA Elektronik, Germany) amplifier with 50 kHz sampling rate and low pass filtered at 3 kHz (Bessel). The slices were placed in the recording chamber filled with aCSF and controlled temperature at 34°C (Scientifica, UK). Electrodes were fabricated from borosilicate glass (BF150-86-10, Sutter Instruments, Novato CA) with tip resistances of 3–5 MΩ.

Glutamatergic excitatory post-synaptic currents (EPSCs) were recorded in the presence of the GABAA antagonist picrotoxin (20 μM). They were evoked stimulating the Schaffer collaterals with a concentric bipolar microelectrode (FHC–Bowdoin, ME, USA), connected to a SD9 Grass voltage stimulator (Natus Medical Incorporated, Warwick, RI, USA). We used the minimum voltage necessary to produce the current with maximum amplitude. EPSCs were recorded at holding potentials from -80 mV to +70 mV, with increments of +30 mV with an internal solution consisting of (mM): 130 CsCl, 10 Hepes, 5 EGTA, 5 phosphocreatine, 4 Mg-ATP, 0.5 Na-GTP, 10 TEA, 5 QX 314, pH adjusted to 7.3 with CsOH and ≈ 290 mOsm/kgH_2_O.

To isolate NMDA currents, we applied the AMPA/kainite antagonist 6,7-dinitroquinoxaline-2,3-dione (DNQX; 10 μM). AMPA/KA currents were obtained by subtracting the currents before and after DNQX, and the remaining currents were mediated by NMDA receptors, confirmed by their sensitivity to the NMDA antagonist DL-AP5. We considered AMPA-kainate receptors fully blocked only when after application of DNQX, we did not observe eEPSCs at -80 mV. Only after this assessment, we recorded the NMDA receptor-mediated currents. The NMDA-AMPA ratio was obtained in the same cell by dividing the current evoked at +70 mV by the current evoked at -80 mV. Short-term depression was evaluated in EPSCs evoked by a train of five stimuli delivered at 20 Hz. In these experiments, we used 6 control animals and 8 stimulated animals.

Spontaneous GABAergic currents were recorded at -80 mV in the presence of DNQX with an internal solution consisting of (mM): 145 KCl, 10 Hepes, 0.5 EGTA, 10 phosphocreatine, 4 Mg-ATP, 0.3 Na-GTP, adjusted to pH 7.3 with KOH and ≈ 290 mOsm/kgH_2_O. Spontaneous inhibitory postsynaptic currents (sIPSCs) were recorded for 10 minutes. We then applied tetrodotoxin (TTX, 0.5μM) to block action potentials and record miniature IPSCs (mIPSCs). In these experiments, we used 7 control animals and 8 stimulated animals.

Series resistance (<20 MΩ) was compensated in 60%. Any neuron with series resistance increased over 20% during experiments were discarded. Voltages were corrected off-line for a liquid junction potential for each internal solution calculated with Clampex software (Molecular Devices).

### Data analysis

Electrophysiological data were analyzed using Mini Analysis (Synaptosoft 6.0.3, Fort Lee, NJ, USA) and custom-written routines in IgorPro (Wavemetrics, Portland, OR, USA) and Matlab (MathWorks, Natick, MA, USA). The peak of the EPSCs was used to build IV relationships to estimate the slope conductances using the inward part of the IV plot for the AMPA/KA current and the outward region for the NMDA currents.

We used GraphPad Prism 6.0 (GraphPad Software, La Jolla CA, USA) for statistical analysis. Histograms were built with the same fixed bins for different groups of cells and fitted with single Gaussian functions. Analysis of decay kinetics for inhibitory currents was performed by Mini Analysis group analysis with individual currents fitted with double exponential functions. Fast and slow time constants were presented as average, and their cumulative distributions were compared. EPSCs decay times were obtained by adjusting the decay phase with a single exponential function, using custom routines.

Data are reported as mean ± SEM. Unpaired t-test was used to compare the means, setting the significance level (p) below 0.05. We used two-way ANOVA to compare the train of EPSCs. Outliers were identified by the ROUT method of nonlinear regression (Q = 1%).

### Drugs

The following drugs were used: picrotoxin (Sigma, 20 μM), DNQX (Sigma, 10 μM) and tetrodotoxin citrate (TTX); Alomone Labs, 0.5 μM). DNQX was dissolved in DMSO and then added to bath from fresh stock solutions. The final concentration of DMSO in the experiments was 0.1%, and we did not find differences in the neurotransmission between DMSO and aCSF. All salts were of reagent grade.

## Results

### High-intensity noise does no change glutamatergic excitatory transmission

**Fig 1:**
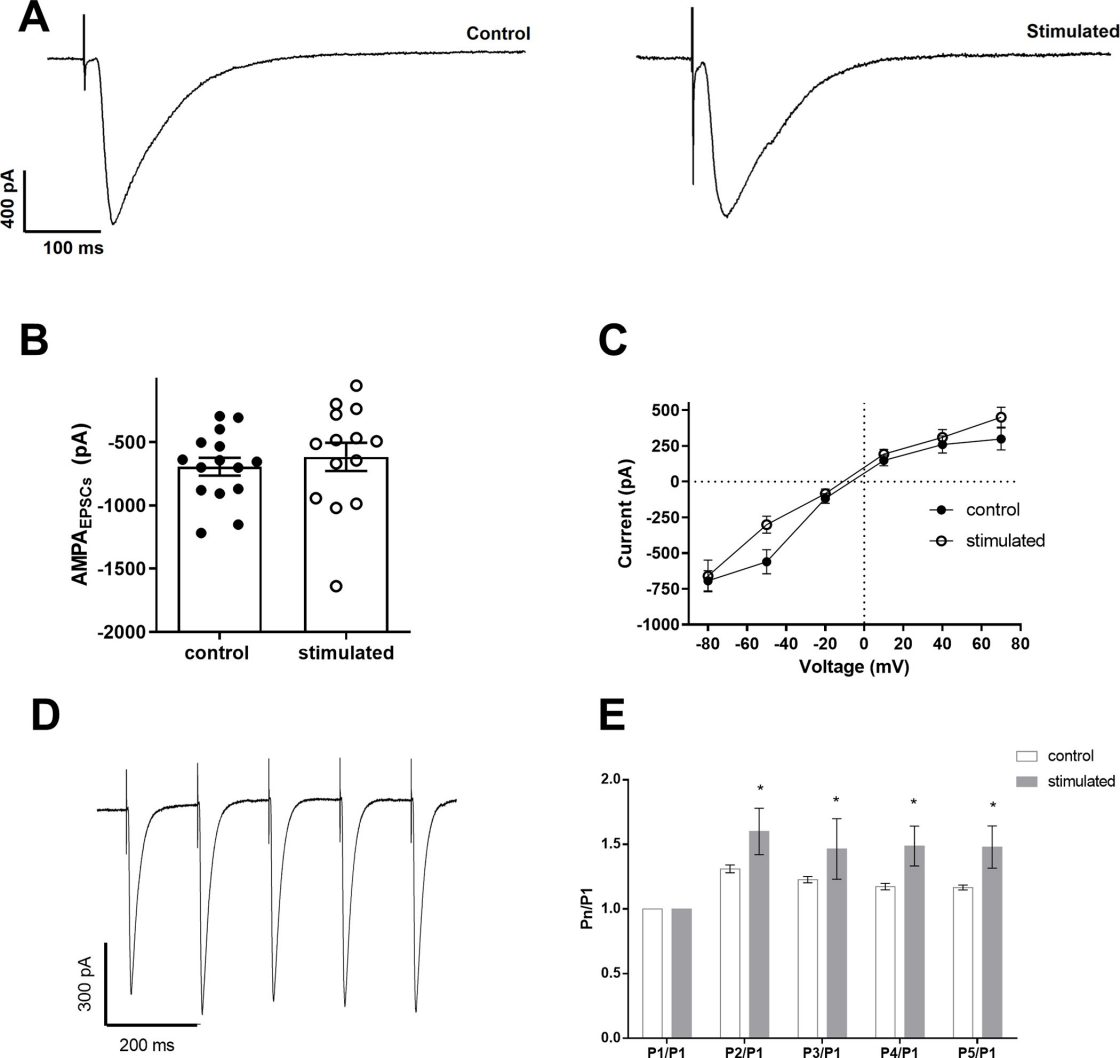
Evoked excitatory AMPA/KA post-synaptic currents (EPSCs) in CA1 pyramidal cells. A. EPSCs recorded in the presence of Picrotoxin (20 μM) at -80 mV from control and stimulated animals. D. Mean AMPA/KA current amplitudes evoked at -80 mV. C. IV relationships for AMPA/KA currents. C. EPSCs in response to a 20 Hz train of stimuli. D. P1/Pn relationships.

We compared the Schaffer-collateral AMPA/KA-mediated excitatory post-synaptic currents in both sham and stimulated groups (Fig 1A). We found that AMPA/KA EPSCs from pyramidal neurons from hippocampi of stimulated animals have similar amplitudes (control = -695 ± 71 pA; stimulated = -617 ± 111 pA; p = 0.42; unpaired t-test, N = 15 and 14, respectively; holding potential of -80 mV) (Fig 1B.) and slope conductances (control = 6.3 ± 0.8 nS; stimulated = 8.1 ± 0.9 nS; p = 0.19; unpaired t-test, N = 15 and 14, respectively). IV relationships of AMPA/KA currents (DNQX-sensitive) were also similar, showing a small inward rectification in both groups (Fig 1C). On the other hand, we found increased facilitation of the EPSCs during trains of stimulation in synapses from stimulated animals (20 Hz; Pn/P1 F(1,24) = 5.3; p = 0.03, Two-Way ANOVA, N = 15 and 8, respectively) (Fig 1D and 1E). We found no alterations in the rise times (control = 5.57 ± 2.25 ms; stimulated = 4.6 ± 1.48 ms; p = 0.29; unpaired t-test, N = 15 and 14, respectively) or decay time constants (control = 12.77 ± 3.74 ms; stimulated = 11.54 ± 2.15 ms; p = 0.29; unpaired t-test, N = 15 and 14, respectively) of AMPA-mediated EPSCs at -80 mV.

**Fig 2:**
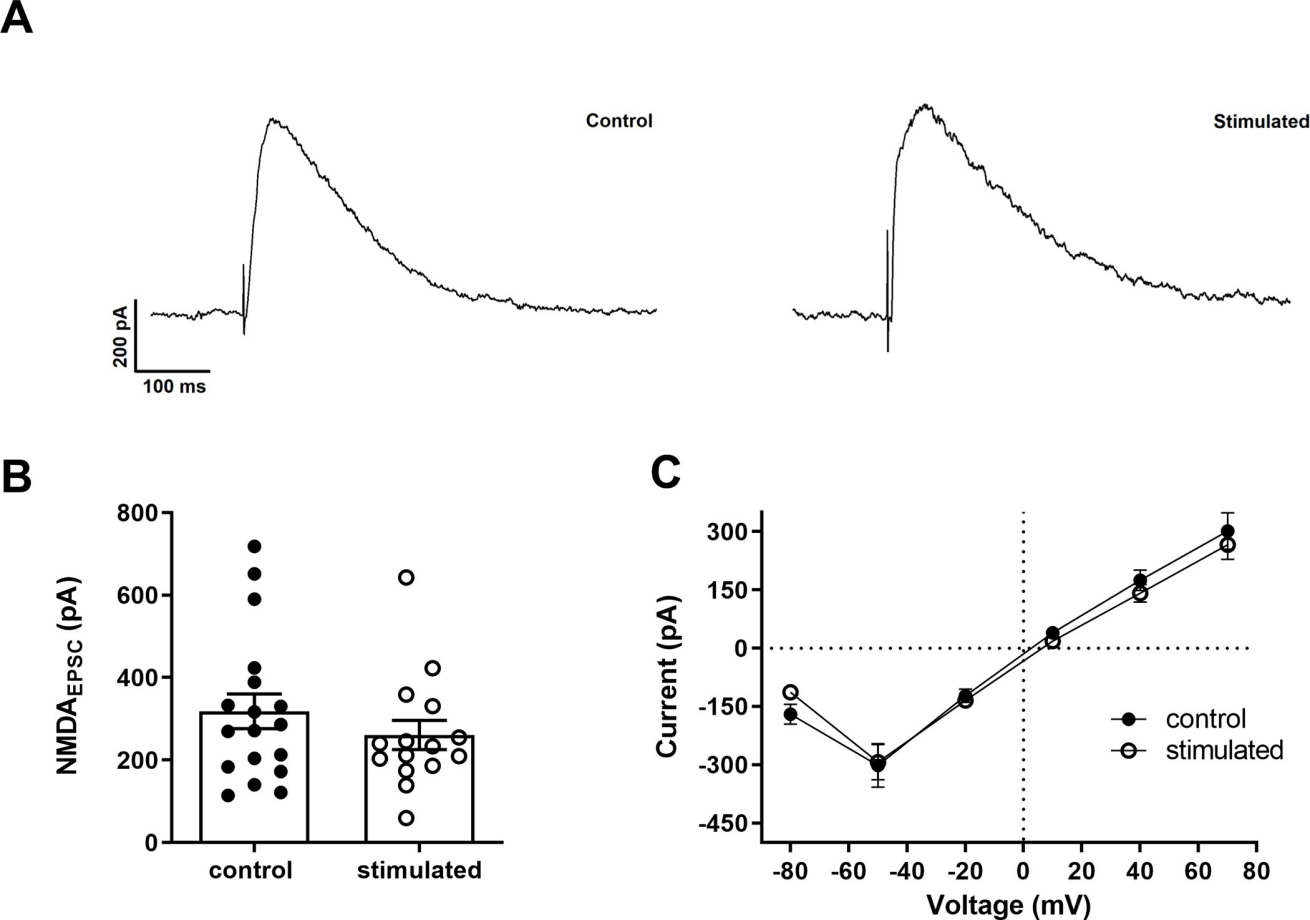
Evoked excitatory NMDA post-synaptic currents (EPSCs) in CA1 pyramidal cells. A. EPSCs recorded in the presence of picrotoxin (20 μM) and DNQX (10 μM) at +50 mV from control and stimulated animals. B. Mean NMDA current amplitudes evoked at +50 mV. C. IV relationships for DNQX-sensitive NMDA currents.

Next, we isolated NMDA-mediated currents using DNQX (10 μM) in order to check whether high-intensity sound could affect these currents, which are crucial for LTP induction in the Schaffer-CA1 synapses (Fig 2A). We found that the amplitudes of NMDA EPSCs were not significantly different in sound stimulated animals (control = 317.5 ± 44.6 pA and stimulated = 261 ± 35.5 pA; p = 0.33; unpaired t-test, N = 17 and 15, respectively. Holding potential of +50 mV; Fig 2B). The IV relationships of the NMDA currents were similar (Fig 2C) and their slope conductances, obtained in the IV were also similar (controls = 4.1 ± 0.8 nS; stimulated = 3.1 ± 0.82 nS; p = 0.42; unpaired t-test, N = 17 and 15, respectively). In addition, we did not observe significant differences in the NMDA/AMPA ratio between cells from controls and stimulated animals (controls = 0.58 ± 0.09 and stimulated = 0.85 ± 0.3; p = 0.4; unpaired t-test, unpaired t-test, N = 17 and 15, respectively). We found no alterations in the rise times (control = 4.57 ± 2.87 ms; stimulated = 5.55 ± 2.32 ms; p = 0.32; unpaired t-test, N = 17 and 15, respectively) or decay time constants (control = 137.2 ± 95.05 ms; stimulated = 106.8 ± 17.3 ms; p = 0.25; unpaired t-test, N = 17 and 15, respectively) of NMDA-mediated EPSCs at +70 mV.

### High-intensity noise potentiates inhibitory GABAergic transmission

**Fig 3:**
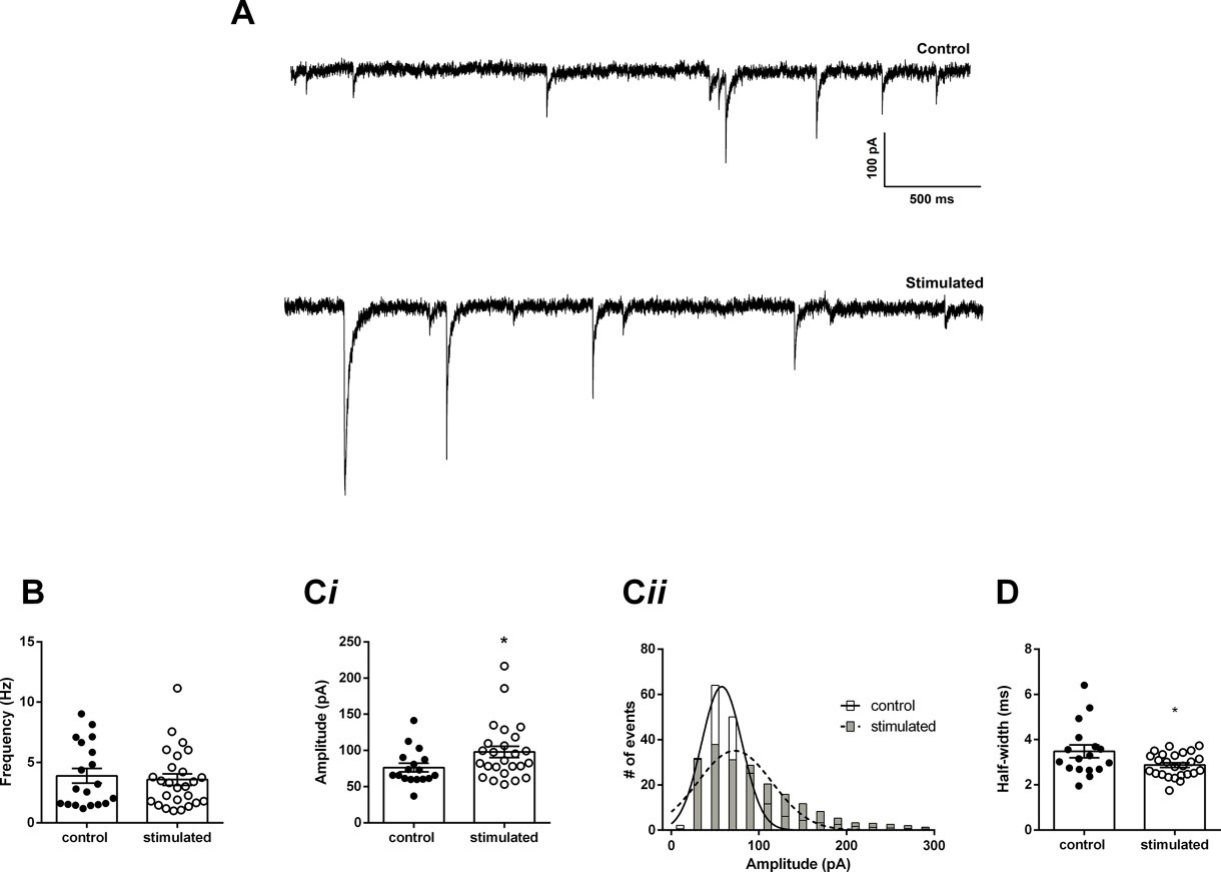
Spontaneous inhibitory post-synaptic currents (sIPSCs) in CA1 pyramidal cells. A. sIPSCs recorded in the presence of DNQX (10 μM) at -80 mV, from control and stimulated animals. B. Mean frequency of IPSCs from controls and stimulated animals. Ci. Mean current amplitudes and, Cii, Amplitude histograms of spontaneous currents from control and stimulated animals (error bars were omitted for clarity). D. Mean half-widths of recorded IPSCs from control and stimulated animals. *p<0.05.

We then investigated the GABAergic transmission on hippocampal CA1 pyramidal neurons, recording the spontaneous GABaergic IPSCs (sIPSCs) in slices from control and stimulated animals (Fig 3A). The frequency of the sIPSCs was not significantly different between neurons from control and stimulated animals (control: 3.9 ± 0.6 Hz; stimulated: 3.6 ± 0.5 Hz; p = 0.7; unpaired t-test, N = 19 and 26, respectively. Fig 3B) but the mean amplitudes were bigger in neurons from stimulated animals (control: -76 ± 4 pA; stimulated: -98 ± 8 pA; p = 0.05; unpaired t-test, N = 17 and 25, respectively. Fig 3C*i*). The distribution of the amplitudes followed a bell-shaped curve with a skewness toward bigger amplitudes (Fig 3C*ii*). Simple Gaussian functions were fitted to the frequency histograms, and they produced curves with significantly different means (control: -57.6 ± 1.5 pA; stimulated: -72.5 ± 2.5 pA; p<0.0001). On the other hand, the half-width of the sIPSCs was shorter in stimulated animals (control: 3.5 ± 0.3 ms; stimulated: 2.8 ± 0.1 ms; p = 0.026; unpaired t-test, N = 17 and 25 respectively. Fig 3D).

**Fig 4:**
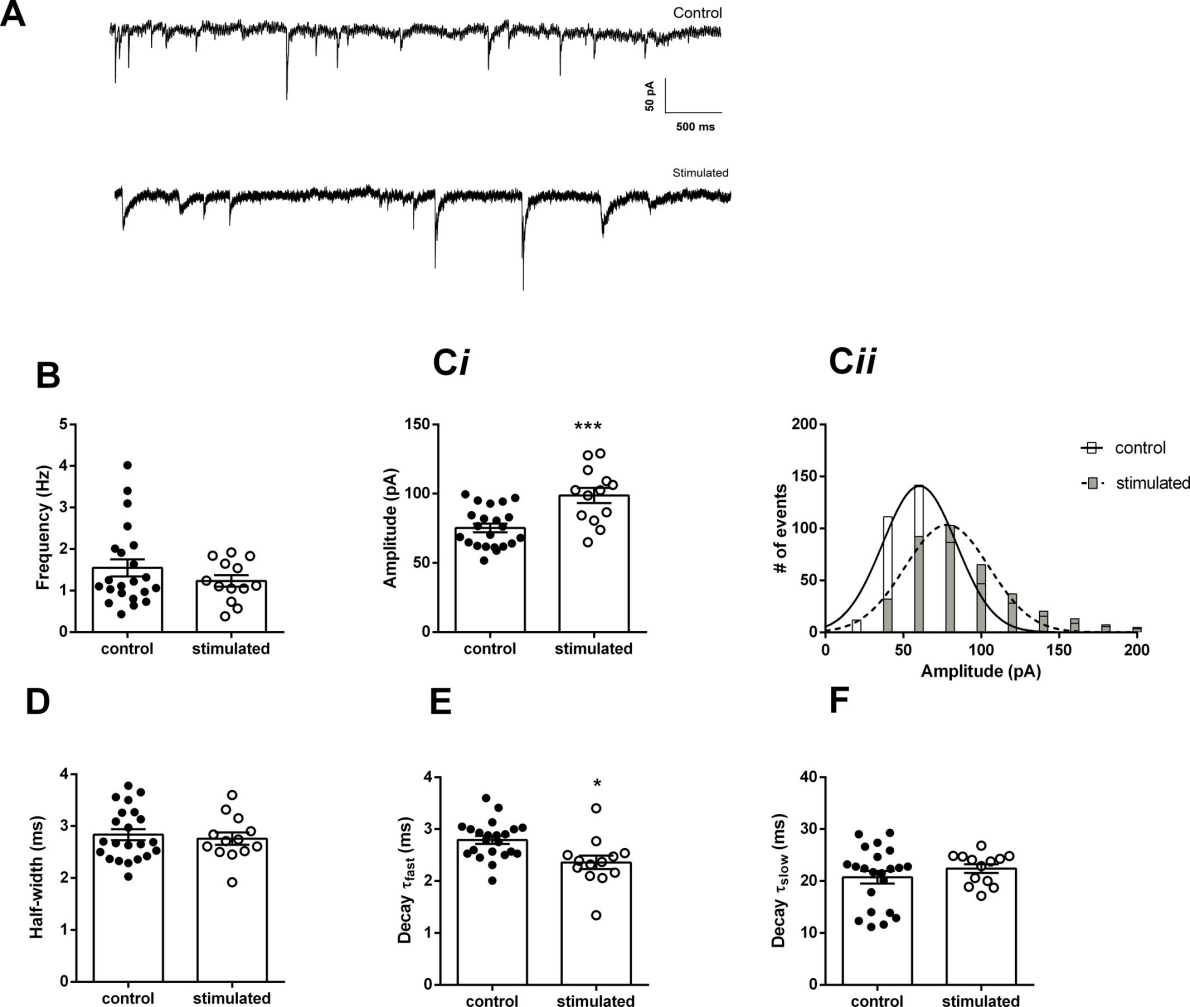
Spontaneous miniature inhibitory post-synaptic currents (mIPSCs) in CA1 pyramidal cells. A. Current traces recorded in the presence of DNQX (10 μM) and TTX (1 μM) at -80 mV, from control and stimulated animals. B. Mean frequency of mIPSCs from controls and stimulated animals. Ci. Mean current amplitudes and Cii, amplitude histograms of spontaneous currents from control and stimulated animals (error bars were omitted for clarity). D. Mean half-widths. E. Mean fast decay time constants. F. Mean slow decay time constants. *p<0.05, ***p<0.001.

Since sIPSCs are affected by the firing of GABAergic neurons, in order to study the properties of GABAergic synapses, we applied TTX to block action potential firing and record the action-potential independent mIPSCs (Fig 4A). Our data show that the frequency of mIPSCs was smaller than the frequency of sIPSCs, reflecting the spontaneous action-potential-independent release of GABA. Again, we did not observe differences in the frequency of mISPCs from control and stimulated animals (control: 1.5 ± 0.2 Hz; stimulated: 1.2 ± 0.1 Hz; p = 0.28; unpaired t-test, N = 22 and 13, respectively. Fig 4B). However, the amplitude of the mIPSCs was significantly bigger in CA1 pyramidal neurons from stimulated animals (control: -75.2 ± 3 pA; stimulated: -98.7 ± 5 pA; p = 0.0003; unpaired t-test, N = 22 and 13, respectively. Fig 4C*i*). The amplitude distribution of the mIPSCs amplitude was fitted with a Gaussian function, and the fits had significantly different means (control: -60.4 ± 1 pA; stimulated: -78.1 ± 1.3 pA; p<0.0001; Fig 4C*ii*). The half-widths were similar (control: 2.8 ± 0.1 ms; stimulated: 2.8 ± 0.1 ms; P = 0.6; N = 22 and 13 respectively. Fig 4D). On the other hand, a more detailed analysis of the decay time of the mIPSCs showed that their fast decay time constant was slightly, but significantly faster in neurons of stimulated animals (control 2.8 ± 0.08 ms; stimulated: 2.4 ± 1.3 ms; p = 0.004; unpaired t-test, N = 22 and 13, respectively. Fig 4E) while the slow decay time constant was not different (control 20.7 ± 1.2 ms; stimulated: 22.4 ± 0.8 ms; p = 0.3; unpaired t-test, N = 22 and 13, respectively. Fig 4F). The fast component comprised of 49.6 ± 0.7% of the total decay time in control animals and 51.9 ± 0.9% in stimulated animals (P = 0.07, unpaired t-test).

## Discussion

Here we showed that GABAergic transmission is potentiated in the CA1 area of the hippocampus of rats after a protocol of 20 episodes of one minute of high-intensity noise, delivered for ten days. On the other hand, we did not observe any change in the excitatory glutamatergic transmission via both AMPA/kainate and NMDA receptors, except an increase in the short-term facilitation during a 20-Hz train. Our results add to others and to our previous findings, showing that the acoustic environment affects the hippocampus.

We have shown previously that the Schaffer-CA1 LTP was inhibited in animals one week after exposure to the same sound protocol used in this investigation [18]. We also observed a diminished h current in CA1 pyramidal neurons, which results in a hyperpolarized membrane and increased membrane time constant [19]. The membrane hyperpolarization could negatively affect action potential firing in response to the train of EPSPs used to induce LTP, affecting the coincidence of pre and post-synaptic firing necessary to produce associative LTP in the hippocampus [22]. On the other hand, the increased membrane time constant can potentially increase dendritic summation of the EPSPs [23, 24], which could have a facilitatory effect on LTP. Therefore, to investigate the synaptic mechanisms of high intensity sound on hippocampal LTP, we studied inhibitory and excitatory synapses on CA1 pyramidal neurons. A decreased glutamatergic transmission could cause the inhibitory effect on LTP via NMDA receptors, which are necessary for inducing associative LTP in the Schaffer-CA1 pathway [22]. However, we did not observe any change in the NMDA receptor-mediated glutamatergic transmission. Moreover, a decreased excitatory neurotransmission through AMPA/kainate receptors could also reduce the probability of inducing LTP, but again, we did not observe any change in this transmission. In contrast, we even found bigger facilitation of the EPSCs during a 20 Hz train. Thus, we cannot explain the decrease in LTP by long-term sound exposure by a reduction in the glutamatergic transmission.

The hippocampus has several types of GABAergic interneurons, which provide strong inhibition controlling the excitability of the pyramidal neurons by both GABA_A_ and GABA_B_ receptors [25, 26]. In hippocampal pyramidal neurons, GABAergic synapses are in dendrites along with the excitatory terminals, in the proximal dendrites, and in cell somata [27]. Activation of dendritic GABAergic synapses increases the threshold for the firing of pyramidal neurons, while the activation of proximal and somatic synapses reduces the maximal firing [26]. Our data show an increase in the amplitude of GABAergic currents after exposure to our high-intensity sound protocol, in both the spontaneous and the action potential-independent miniature IPSCs. The lack of change in the frequency and the persistence of this effect after perfusion of TTX strongly suggests that these effects are post-synaptic, on the GABA_A_ receptor level, and not on the release probability of GABAergic vesicles or interneuron firing. The small change in the decay kinetics of the mIPSCs from stimulated animals suggests a shift in receptor composition.

These stronger GABAergic currents could reduce the excitability of the pyramidal neuron during the induction of LTP reducing its firing and impairing LTP induction, which depends upon pre-and post-synaptic firing. Somatic inhibition is more effective to diminish pyramidal neuron firing than dendritic inhibition [26], and most of our recorded spontaneous GABAergic currents are probably of somatic origin because dendritic currents are primarily filtered by the dendritic cable resistance [28]. Interestingly, dendritic GABAergic mIPSCs are much more sensitive to TTX than somatic mIPSCs [29] suggesting that our mIPSCs were also of somatic origin. Additionally, somatic whole cell recording seems to increase the probability of GABA release from somatic synapses increasing the chance to record somatic IPSCs and mIPSCs [30].

An increase in GABAergic neurotransmission could have a protective effect against the development of audiogenic limbic seizures since GABAergic inhibition controls the excitability of the hippocampal circuit and a failure in GABAergic inhibition may lead to the development of hippocampal seizures [31]. Interestingly, GABAergic inhibition on CA1 pyramidal neurons is decreased in the hippocampus of a strain susceptible to audiogenic seizures [32].

This increased GABAergic tone is also a potential contributor to the inhibited LTP after high-intensity sound stimulation; although, at this moment, we cannot make any causal conclusion about the mechanism of LTP inhibition. It is possible that this effect is a compensatory mechanism for the increased firing of the pyramidal neurons after high-intensity sound stimulation, for instance [19]. More studies are being carried out to elucidate the mechanism of LTP inhibition by high-intensity sound exposure. Nevertheless, our findings show that the hippocampal inhibitory neurotransmission is affected by high-intensity sound exposure, which can have relevant consequences to the hippocampal function in animals and humans subjected to constant or even episodic loud sound exposure in their environments.
